# Extended-Spectrum Beta-Lactamase (ESBL)-Producing *Escherichia coli* Isolated from Flies in the Urban Center of Berlin, Germany

**DOI:** 10.3390/ijerph16091530

**Published:** 2019-04-30

**Authors:** Wibke Wetzker, Yvonne Pfeifer, Solvy Wolke, Andrea Haselbeck, Rasmus Leistner, Axel Kola, Petra Gastmeier, Florian Salm

**Affiliations:** 1 Institute of Hygiene and Environmental Medicine, Charité—Universitätsmedizin, 12203 Berlin, Germany; solvy.wolke@charite.de (S.W.); Andrea.Haselbeck@ivi.int (A.H.); rasmus.leistner@charite.de (R.L.); axel.kola@charite.de (A.K.); petra.gastmeier@charite.de (P.G.); florian.salm@charite.de (F.S.); 2 National Reference Center for the Surveillance of Nosocomial Infections, Charité—Universitätsmedizin, 12203 Berlin, Germany; 3 Robert Koch Institute, FG13 Nosocomial Pathogens and Antibiotic Resistance, 38855 Wernigerode, Germany; PfeiferY@rki.de; 4 International Vaccine Institute, Seoul 08826, Korea

**Keywords:** antimicrobial resistance, multidrug resistance, CTX-M-1, plasmid transfer, surveillance

## Abstract

*Background*: The monitoring of antimicrobial resistance (AMR) in microorganisms that circulate in the environment is an important topic of scientific research and contributes to the development of action plans to combat the spread of multidrug-resistant (MDR) bacteria. As a synanthropic vector for multiple pathogens and a reservoir for AMR, flies can be used for surveillance. *Methods*: We collected 163 flies in the inner city of Berlin and examined them for extended-spectrum β-lactamase (ESBL)-producing *Escherichia coli* genotypically and phenotypically. *Results*: The prevalence of ESBL-producing *E. coli* in flies was 12.9%. Almost half (47.6%) of the ESBL-positive samples showed a co-resistance to ciprofloxacin. Resistance to carbapenems or colistin was not detected. The predominant ESBL-type was CTX-M-1, which is associated with wildlife, livestock, and companion animals as a potential major source of transmission of MDR *E. coli* to flies. *Conclusions*: This field study confirms the permanent presence of ESBL-producing *E. coli* in an urban fly population. For continuous monitoring of environmental contamination with multidrug-resistant (MDR) bacteria, flies can be used as indicators without much effort.

## 1. Introduction

The rise of antibiotic-resistant Gram-negative bacteria poses a serious threat to health, food security, and prosperity on a global scale. Monitoring reports demonstrated that the prevalence of extended-spectrum β-lactamase (ESBL)-producing *Enterobacteriaceae* resistant to penicillins and third generation cephalosporins has been increasing significantly since the turn of the millennium [1,2,3,4]. Particular attention is paid to *Escherichia coli*, a common cause of urinary tract, wound, and bloodstream infections. Its omnipresence in various habitats and its ability to acquire resistance genes via mobile genetic elements enables *E. coli* to rapidly disseminate antimicrobial resistance (AMR) between humans, animals, and the environment. To understand the pathways connecting different reservoirs of ESBL-producing *E. coli* and to learn about the factors having an impact on colonization and transmission in humans and animals, the surveillance of resistant *E. coli* from the environment and different populations might offer a missing piece to the AMR puzzle. Studies on livestock and companion animals confirmed the wide spread of various ESBLs in *E. coli*, and findings on the presence of ESBL-producing *E. coli* in wild animals, wastewater, and surface waters are broadening the picture [5,6,7,8].

Synanthropic animals cohabiting with humans show a high potential as vectors for the transmission of pathogens and resistances. Insects work as environmental proxies that might indicate areal contamination with multidrug-resistant (MDR) bacteria, such as ESBL-producing *E. coli* [9,10,11,12]. Synanthropic flies in particular can reflect the resistome of their habitat as they feed and breed on organic material such as garbage, wastewater, feces, and carrion. MDR bacteria can colonize the intestines of filth flies and horizontal transfer of AMR genes in the alimentary canal has been reported [13,14,15]. The prevalence of *E. coli* isolated from flies varies between 10.5% and 76.3% globally [16]. A single study of flies in Germany in 2011 revealed that 27% of collected samples carried one or more strains of *E. coli* [17]. We conducted a cross-sectional study in the urban center of Berlin in 2016 to gain data on the current prevalence of ESBL-producing *E. coli* in flies.

## 2. Materials and Methods

Flies were collected in July 2016 under meteorologically stable conditions without rainfall and with daytime outside temperatures between 20 and 30 °C. The study population included any synanthropic filth flies (order Diptera) that do not feed on blood or pollen exclusively. As with flies, the composition of the microbiome depends not so much on the species but on the habitat, food, and breeding grounds; therefore, flies were examined in groups depending on the site of sampling [18,19]. The samplings were conducted at four sites within a quadrant of 5 × 5 km in the city center of Berlin: the disposal area of a kitchen and catering service on the premises of a tertiary hospital (site H), the fence of an open air zoo (site Z), and two streets in randomly selected residential areas (site RA and RB). All sites showed basic characteristic similarities (potential feeding sources like waste or animal feces in sight), and were located in the immediate vicinity of an urban waterway.

A target sample size of 163 was analyzed using the free software OpenEpi (https://www.openepi.com/Menu/OE_Menu.htm) to give a mean prevalence of ESBL-producing *E. coli* in the fly population—12% according to published data [11,14,20,21]—and a confidence level of 95%. At all four sites, self-built funnel traps made from commercially available PET water bottles (1.5 liters) were placed outdoors (https://www.wikihow.com/Catch-Small-Bugs#/Image:Catch-Small-Bugs-Step-9.jpg). Sticky traps were avoided with regard to previously reported bias [22]. As bait, a tablespoon of wet cat food or sheep’s rumen was placed at the bottom of the trap. Furthermore, a sample was taken from each bait and tested for the presence of ESBL-producing bacteria to exclude cross-contamination. Within 24 h after placing a single trap, ten to twenty live flies were taken from each trap and placed into commercial plastic bags for transportation to the laboratory. Within the residential areas, flies were also individually caught using a sweep net indoors. Flies were transported to the Institute of Hygiene and Environmental Medicine, Charité Universitätsmedizin, Berlin, for further analyses.

All flies were identified at the family level, but were not examined for species or sex. For the dissection of the flies, we followed the main steps of a standardized protocol Pava-Ripoll and colleagues developed for the detection of bacterial pathogens from individual filth flies [23]. Flies were immobilized at minus 80 °C for up to five minutes and then killed and surface sanitized in polystyrene conical tubes (Falcon) filled with 10 mL of 80% ethanol to rule out cross-contamination within the traps. This procedure is based on the fact that most bacterial species could be detected on the exoskeleton and in the alimentary canal of flies; it is known that diversity and concentration are higher in the digestive tract than on the body’s surface [24,25,26,27]. The use of ethanol does not distort the highly complex microflora of the fly significantly [28]. After the decantation of ethanol, the flies were washed individually in sterile, physiological saline solution (NaCl 0.9%, Braun, Melsungen, Germany), and were transferred to Petri dishes (Cell culture dish 100 × 20 mm, Sarstedt AG, Nümbrecht, Germany) for drying. Each fly was transferred to a tube containing 8 mL of pre-warmed (37 °C) BBL Trypticase Soy Broth (Becton, Dickinsion and Co., Heidelberg, Germany) and homogenized. The tubes with the suspension were incubated at 35 °C to 37 °C for 24 h.

For isolation and phenotypical identification of ESBL-producing *E. coli*, the suspension was cultured on Columbia blood agar (BioMerieux, Nürthingen, Germany) as a non-selective medium, on MacConkey agar (BioMerieux) as a selective medium for Gram-negative bacteria, and on selective chromogenic medium for the screening of ESBL-producers (BioMerieux) in two fractions per incubated tube (half a plate per sample). The plates were incubated at 37 °C. Growth was monitored after 24 and 48 h. VITEK^®^ 2 Compact automated system (GN card; AST-N223-card, BioMerieux, Nürthingen, Germany,) was used to verify ESBL-producing *E. coli* and to detect antimicrobial susceptibilities using EUCAST 2016 and CLSI 2016 breakpoints for the interpretation of results. When morphologically different ESBL-producing *E. coli* colonies were observed in one sample, it was counted once as “ESBL positive” for the statistical calculation of prevalence. For statistical analyses, open source software OpenEpi and R (Dormagen, Germany, https://www.r-statistik.de/) was used.

All isolates with a resistance to cefotaxime and/or ceftazidime and positive ESBL confirmation disk test (ESBL/AmpC detection disk set D68C, MAST Diagnostica GmbH, Reinfeld, Germany) were sent to the Robert Koch Institute for molecular analyses. Presence of different β-lactamase genes (*bla*_TEM-like_, *bla*_SHV-like_, *bla*_CTX-M-1-2-8-9-25group_, *bla*_CMY-like_, *bla*_KPC-like_, *bla*_VIM-like_ *bla*_OXA-48-like_, and *bla*_NDM-like_), and genes contributing to fluoroquinolone resistance (*qnrA/B/S*, *aac*(*Ib*)-*cr*) was tested using PCR and Sanger sequencing (Supplementary Table S1). Furthermore, the potential presence of plasmid-mediated colistin resistance genes (*mcr-1-like* to *mcr-8-like*) and the previously described association of *bla*_LAP_ and PMQR gene *qnrS* was tested using PCR (Table S1). The phylogenetic grouping of *E. coli* isolates and determination of the proportion of the common *E. coli*-sequence type (ST)131 was done using PCR-based assays [29,30]. The further genetic relationship of the isolates was evaluated using XbaI macrorestriction and subsequent pulsed-field gel electrophoresis (PFGE) with an interpretation according to the criteria of Tenover et al. [31]. The transfer of third generation cephalosporin resistance was tested in broth mate conjugation assays using an *E. coli* J53 Azi^R^ recipient and selective Luria-Bertani agar plates with 200 mg/L sodium azide and 1 mg/mL cefotaxime. If ESBL genes were transferred, plasmid sizes were determined via S1-nuclease restriction and PFGE [32].

## 3. Results

Samples were classified as blowflies (Diptera: Calliphoridae), houseflies (Diptera: Muscidae), or flesh flies (Diptera: Sarcophagidae). The total sample size of *n* = 163 was not equally distributed across the four sampling sites. In the residential areas (RAs), significantly fewer flies were caught than in other areas (26/163; Table 1). ESBL-producing *E. coli* were phenotypically verified in 21 of the 163 flies (12.9%; Table 1). All cultures of bait samples showed bacterial growth, but ESBL-producing *Enterobacteriaceae* were not detected. Also, all homogenized fly samples showed bacterial growth on the non-selective medium and on the selective medium for Gram-negative bacteria. 

Using the Pearson chi-square test, there was no significant association (significance level 0.05) between the site and the presence of ESBL-producing *E. coli* in the fly sampling (χ^2^ = 4.439; d = 3; *p* = 0.218). Since for residential area A, an expected value of <5 ESBL-positive flies (3.35) was found in the contingency table, a robust method (Monte Carlo simulation) was used to confirm the results from the chi-square test (*p* = 0.223).

In ten fly samples that had been confirmed with ESBL-producing *E. coli* (6%), a co-resistance to fluoroquinolones (ciprofloxacin) was detected. Five ESBL-positive fly samples (3%) showed additional non-susceptibility to the group of folic acid antagonists (trimethoprim/sulfamethoxazole). No resistances to carbapenems or colistin were detected. Genes encoding CTX-M-type ESBLs were identified in 22 out of 24 ESBL-producing *E. coli* (92%) isolated from 21 fly samples, and CTX-M-1 was the most prevalent type (15/24; Table 2). The genes *bla*_CTX-M-15_, *bla*_CTX-M-14_, *bla*_SHV-12_ and bla_CTX-M-3_ were detected in four, two, two, and one isolates, respectively. Furthermore, the plasmid-mediated gene *qnrS1* contributing to quinolone resistance was found in 13 of the 24 isolates. Using PCR-based assays, the 24 ESBL-producing *E. coli* isolates could be assigned to phylogenetic groups A (14 isolates), D (8 isolates), and B1 (2 isolates). No *E. coli*-ST131 was confirmed using PCR. PFGE typing revealed that the 24 isolates belonged to 14 different *E. coli* clones, whereby the same clones occurred only at the same sampling sites (Table 2). For seven of the 14 *E. coli* clones, conjugative transfer of the ESBL genes (5 *bla*_CTX-M-1_, 1 *bla*_SHV-12_, and 1 *bla*_CTX-M-3_) was successful; co-transfer of *qnrS1* was observed for two clones. The ESBL gene-carrying plasmids were of various sizes (35kb–160kb, Table 2, Figure S1).

## 4. Discussion

The present study analyzed genotypical and phenotypical characteristics of ESBL-producing *E. coli* in flies collected in an urban area in Germany. All flies from our sampling were assigned to the families Calliphoridae, Muscidae and Sarcophagidae that are well studied as vectors for various potential pathogens and/or reservoirs of AMR [16,33,34]. The identified ESBL prevalence rate of 12.9%; (*n* = 163) showed no significant difference compared to the results from two earlier studies in the Netherlands, which examined flies on poultry farms in 2011 (10.5%, *n* = 87) and 2012 (15.0%, *n* = 73); (χ^2^ = 0.127; d = 1; *p* = 0.722) [11,21]. Under careful consideration of deviating geographical location and a time interval of five years, flies from rural and urban areas seem equally affected by the spread of AMR.

Our results support the findings of previous studies that associated synanthropy with the presence of MDR *Enterobacteriaceae* not only in livestock and companion animals, but also in rats, gulls and insects [35,36,37]. However, ESBL prevalence rates and antibiotic susceptibilities can vary significantly by sampling site, which implies geographical clustering that needs to be considered to a local scale [38,39].

For interspecific comparison there is data available from a similar study carried out in Berlin in 2010. 56 brown rats were screened for ESBL-producing *E. coli* and a prevalence of 16% was determined [36]. In hospitalized patients colonization rates of ESBL-producing *E. coli* was slightly below the values reported for rats and flies (11.7% on admission) in a study conducted at the Jena University Hospital between 2013 and 2015 [40]. However, a prevalence of ESBL-producing *E. coli* up to 70% was detected in samples taken from 150 German livestock farms [41]. Especially in cattle and pigs, but also in poultry, zoo and companion animals CTX-M-1 was the most frequent ESBL type [42,43]. A commonly identified pattern containing ESBL gene blaCTX-M-1 in combination with *E. coli* of phylogenetic group A was also confirmed to be predominant for flies in our study [41,42]. 

By contrast, infections with ESBL-producing *E. coli* in humans are predominantly associated with ESBL-type CTXM-15 (>50%) and *E. coli* of phylogenetic group B2, with *E. coli* O25b:H4-ST131 being the most common clonal lineage (up to 40% of all ESBL-producing *E. coli*) [40,44,45]. Neither *E. coli*-ST131 nor isolates of phylogenetic group B2 were confirmed in our study. However, these studies also report that 25–30% of the ESBL-producing *E. coli* from hospitalized patients and outpatients are CTX-M-1 producers assigned to *E. coli* of phylogenetic groups A and D (7–30%) [45]. We assume that animals or their feces as primary sources of transmission of MDR *E. coli* to flies but also excrements of colonized humans may play a role. Feed or water sources such as waste and sewage are worth considering [46,47,48,49,50]. In a Spanish study the plasmid mediated gene *qnrS* that contributes to quinolone resistance was found to be associated with urban wastewater samples [51]. We confirmed *qnrS* in 54.2% (13/24) of our ESBL-producing *E. coli* isolated from flies.

Results from the molecular analysis coincide to large extend with those from a study conducted in the urban and rural areas near the city of Munster (450 km West of Berlin) in the summer of 2015: Schaumburg et al. reported CTX-M-1 to be the most prevalent β-lactamase in isolates of ESBL-producing *E. coli* in flies [39]. Phylogenetic group A was detected in 79.6% of isolates (in our study 58.3%) and phylogenetic group B2 was not confirmed for flies. In both studies phylogenetic group D isolates were the second most frequent. While phylogenetic group A and B1 usually are associated with commensal *E. coli* strains, phylogenetic group D is associated with more virulent extra-intestinal strains and infections in humans [52]. Data from Munster and Berlin differed with regards to ESBL gene *bla*_CTX-M-14_ that was found in two samples (1 *E. coli* clone) but not in Munster. Studies showed low prevalence of CTX-M-14 in livestock in Europe (4–7%) [42,44]. Bacterial strain typing and conjugation experiments on our study isolates demonstrated a high diversity of different *E. coli* clones in flies that had acquired different ESBL genes, mainly *bla*_CTX-M-1_, that were located on plasmids of variable size. Further and deeper investigations, e.g. whole genome sequencing analyses, are necessary for a better understanding of the environmental contamination by AMR and the pathways of transmission.

The present work has some methodological limitations. The target sample size of *n* = 163 was not equally distributed across the sampling sites. A deviating prevalence of ESBL-producing *E. coli* between the two residential areas (RA 0%, *n* = 26; RB 19.1%, *n* = 42) is noticeable, yet no significant difference was statistically proven. A possible explanation for the absence of ESBL-producing *E. coli* in residential area A (RA) is the fact that most of the flies were caught in relatively closed interiors (apartments that are regularly ventilated). In contrast residential area B (RB) was characterized by backyards with green areas where much of the flies were caught. Outdoor flies are more likely to be in contact with known reservoirs of ESBL-producing *E. coli* such as dog feces [53]. Increasing the sample size and the spatial and taxonomic resolution may influence the study results.

## 5. Conclusions

We confirmed the presence of ESBL-producing *E. coli* in 12.9% of the flies caught in the city of Berlin. We identified predominantly plasmid-encoded ESBL-type CTX-M-1 and *E. coli* of phylogenetic group A. Isolates could not be linked to a specific source, but we considered an animal origin, presumably livestock, zoo, or companion animals. Our study strengthens the scientific assumption of a progressing environmental pollution via AMR that refers to a common source from humans and/or animals and extends across multiple routes of dissemination. Further investigations of the urban resistome seem to be necessary and whole genome sequencing would be the method of choice. Future research should focus on the acquisition of complementary data from environmental, veterinary, and human samples. Surveillance of AMR in *Enterobacteriaceae* should be established as an integral part of a global public health policy.

## Figures and Tables

**Table 1:** Prevalence of ESBL-producing *E. coli* in 163 flies caught in Berlin, Germany.

Site	*n*	ESBL-Positive	%	CI *
H	45	6	13.3	6.3–26.2
Z	50	7	14.0	7.0–26.2
RA	26	0	0	0–12.9
RB	42	8	19.1	10.0–33.3
total	163	21	12.9	8.6–18.9

* Confidence interval 95%; Sites: H—hospital, Z—zoo, RA—residential area A, RB—residential area B.

**Table 2:** Molecular characteristics of 24 ESBL-producing *E. coli* isolates from 21 flies.

Sample No. and Site	Isolate No.	ESBL	Other β-Lactamases	PMQR Genes	Phylogenetic Group	PFGE Type (Clone)	Plasmid Size *
20 H	752/17	CTX-M-1	-	-	A	E1	80 kb
31 H	753/17	SHV-12	-	*qnrS1*	A	E2	35 kb
32 H	754/17	CTX-M-1	TEM	-	A	E3	160 kb
48 H	755/17	SHV-12	-	*qnrS1*	A	E2	35 kb
52 H	756/17	CTX-M-15	-	-	B1	E4	n.t.
55 H	757/17	CTX-M-15	-	-	B1	E4	n.t.
61 Z	758/17	CTX-M-1	-	-	A	E5	90 kb
62 Z	759/17	CTX-M-3	-	-	D	E6	80 kb
69 Z	760/17	CTX-M-14	-	-	D	E7	n.t.
71 Z	761/17	CTX-M-14	-	-	D	E7	n.t.
73 Z	762/17	CTX-M-1	-	-	A	E8	n.t.
79 Z	763/17	CTX-M-1	-	-	D	E9	105 kb
84 Z	764/17	CTX-M-15	-	-	D	E10	n.t.
122 RB	765/17	CTX-M-15	TEM	*qnrS1*	A	E11	n.t.
146 RB	766/17	CTX-M-1	TEM	*qnrS1*	D	E12	n.t.
153 RB	767/17	CTX-M-1	TEM, LAP	*qnrS1*	A	E13	n.t.
157 RB	768/17	CTX-M-1	LAP	*qnrS1*	A	E14	50 kb
157 RB	769/17	CTX-M-1	LAP	*qnrS1*	A	E14	50 kb
160 RB	770/17	CTX-M-1	TEM	*qnrS1*	A	E13	n.t.
163 RB	771/17	CTX-M-1	LAP	*qnrS1*	A	E14	50 kb
163 RB	772/17	CTX-M-1	TEM	*qnrS1*	D	E12	n.t.
165 RB	773/17	CTX-M-1	LAP	*qnrS1*	A	E14	50 kb
165 RB	774/17	CTX-M-1	TEM	*qnrS1*	D	E12	n.t.
166 RB	775/17	CTX-M-1	LAP	*qnrS1*	A	E14	50 kb

Sites: H—hospital, Z—zoo, RA—residential area A, RB—residential area B; PMQR, plasmid mediated quinolone resistance; * plasmids containing the ESBL gene, transferred using broth mate conjugation assay in *E. coli* J53 Azi^R^; plasmid size was determined using S1-nuclease-PFGE analysis (see Table S1); n.t. not tested—transfer of resistance by both mate conjugation was not successful; PCR amplicons of *bla*_TEM-like_ and *bla*_LAP-like_ genes were not sequenced.

## Supplementary Materials

The following are available online at https://www.mdpi.com/1660-4601/16/9/1530/s1, Table S1: Primer sequences for PCR screening and Sanger sequencing; Figure S1: S1-nuclease restriction and pulsed-field gel electrophoresis (PFGE): plasmids of donor *E. coli* isolates from flies and their transconjgants.
