# Supplementary material for: Extended-Spectrum Beta-Lactamase (ESBL)-Producing Escherichia coli Isolated from Flies in the Urban Center of Berlin, Germany
Source: Int J Environ Res Public Health. 2019 Apr 30;16(9):1530. doi: 10.3390/ijerph16091530 (PMC6539871; doi:10.3390/ijerph16091530)
Supplement: Supplementary file 1 [file ijerph-16-01530-s001.pdf]

International Journal of
Environmental Research and Public Health

Supplementary Materials:

Table 1. Primer sequences for PCR screening and Sanger sequencing.

Primer name	Resistance genes	Sequence 5′---3′	Product length	Reference
TEM u FWD<br>TEM u REV	*bla*TEM	ATGAGTATTCAACATTTCCG<br>TTAATCAGTGAGGCACCTAT	851 bp	1
SHV u FWD<br>SHV u REV	*bla*SHV	GCAAAACGCCGGGTTATTC<br>GGTTAGCGTTGCCAGTGCT	940 bp	1
CTX-M-9 FWD<br>CTX-M-9 REV	*bla*CTX-M-9-group	GCAGTACAGCGACAATACCG<br>TATCATTGGTGGTGCCGTAG	356 bp	1
CTX-M-u FWD<br>CTX-M-u REV	*bla*CTX-M-1&2-group	CGCTTTGCGATGTGCAG<br>ACCGCGATATCGTTGGT	551 bp	1
CTX-M-ges FWD<br>CTX-M-ges REV	*bla*CTX-M-1-group	GTTCGTCTCTTCCAGAATAAGG<br>CAGCACTTTTGCCGTCTAAG	968 bp	2
CTX-M-21 FWD<br>CTX-M-21 REV	*bla*CTX-M-9-group	ACACGGATTGACCGTATTGG<br>ATGATTCTCGCCGCTGAAG	889 bp	This study
CTX-M-8 FWD<br>CTX-M-8 REV	*bla*CTX-M-8-group	TAACGCACAGACGCTCTACC<br>TGGCTGGGTGAAGTAAGTGAC	637 bp	4
CTX-M-25 FWD<br>CTX-M-25 REV	*bla*CTX-M-25-group	GCGATGTTAATGACGACAGC<br>AACCGTCGGTGACAATTCTG	847 bp	4
NDM FWD<br>NDM REV	*bla*NDM	CTGAGCACCGCATTAGCC<br>GGGCCGTATGAGTGATTGC	754 bp	3
VIM u FWD<br>VIM u REV	*bla*VIM	AGTGGTGAGTATCCGACAG<br>ATGAAAGTGCGTGGAGAC	261 bp	1
KPC u FWD<br>KPC u REV	*bla*KPC	CAGCTCATTCAAGGGCTTTC<br>AGTCATTTGCCGTGCCATAC	533 bp	1
OXA-48 FWD<br>OXA-48 REV	*bla*OXA-48	AAATCACAGGGCGTAGTTGTG<br>GACCCACCAGCCAATCTTAG	555 bp	1
CMY FWD<br>CMY REV	*bla*CMY	TCTGCTGCTGACAGCCTCT<br>CTCGACACGGACAGGGTTAG	1147 bp	4
QnrA FWD<br>QnrA REV	*qnrA*	ATTTCTCACGCCAGGATTTG<br>GGCAAAGGTTAGGTCACAG	513 bp	2
QnrB FWD<br>QnrB REV	*qnrB*	ATGACGCCATTACTGTATAA<br>GATCGCAATGTGTGAAGTTT	562 bp	2

QnrS FWD<br>QnrS REV	*qnrS*	GGCACCACAACTTTTCAC<br>CAACAATACCCAGTGCTTCG	619 bp	2
AAC(6′)-Ib-cr FWD<br>AAC(6′)-Ib-cr REV	*aac*-like	TTGCGATGCTCTATGAGTGGCTA<br>CTCGAATGCCTGGCGTGTTT	482 bp	5
LAP FWD<br>LAP REV	*bla*$_{LAP}$	CAATACAAAGCACAGAAGACC<br>CCGATCCCTGCAATATGCTC	858 bp	6
Mcr-1 FWD<br>Mcr-1 REV	*mcr-1*	CGGTCAGTCCGTTTGTTC<br>CTTGGTCGGTCTGTAGGG	309 bp	7
Mcr-2 FWD<br>Mcr-2 REV	*mcr-2*	TGTTGCTTGTGCCGATTGGA<br>AGATGGTATTGTTGGTTGCTG	568 bp	8
Mcr-3 FWD<br>Mcr-3 REV	*mcr-3*	TTGGCACTGTATTTTGCATTT<br>TTAACGAAATTGGCTGGAACA	542 bp	9
Mcr-4 FWD<br>Mcr-4 REV	*mcr-4*	ATTGGGATAGTCGCCTTTTT<br>TTACAGCCAGAATCATTATCA	487 bp	10
Mcr-5 FWD<br>Mcr-5 REV	*mcr-5*	TATCTCGACAAGGCCATGCTG<br>GAATCTGGCGTTCGTCGTAGT	613 bp	11
Mcr-6 FWD<br>Mcr-6 REV	*mcr-6*	GTCCGGTCAATCCCTATCTGT<br>ATCACGGGATTGACATAGCTAC	556 bp	12
Mcr-7 FWD<br>Mcr-7 REV	*mcr-7*	TGCTCAAGCCCTTCTTTTCGT<br>TTCATCTGCGCCACCTCGT	892 bp	12
Mcr-8 FWD<br>Mcr-8 REV	*mcr-8*	AACCGCCAGAGCACAGAATT<br>TTCCCCCAGCGATTCTCCAT	667 bp	13

References Table S1

1. Gröbner, S.; Linke, D.; Schutz, W.; Fladerer, C.; Madlung, J.; Autenrieth, I. B.; Witte, W.; Pfeifer, Y., Emergence of carbapenem-non-susceptible extended-spectrum beta-lactamase-producing Klebsiella pneumoniae isolates at the university hospital of Tübingen, Germany. J Med Microbiol 2009, 58, (Pt 7), 912-22.
2. Pfeifer, Y; Matten, J; Rabsch, W., Salmonella enterica serovar Typhi with CTX-M beta-lactamase, Germany. Emerg Infect Dis. 2009, 15, (9), 1533-5.
3. Pfeifer, Y.; Wilharm, G.; Zander, E.; Wichelhaus, T. A.; Gottig, S.; Hunfeld, K. P.; Seifert, H.; Witte, W.; Higgins, P. G., Molecular characterization of blaNDM-1 in an Acinetobacter baumannii strain isolated in Germany in 2007. J Antimicrob Chemother 2011, 66, (9), 1998-2001.
4. Eller, C.; Simon, S.; Miller, T.; Frick, J. S.; Prager, R.; Rabsch, W.; Guerra, B.; Werner, G.; Pfeifer, Y., Presence of beta-lactamases in extended-spectrum-cephalosporin-resistant Salmonella enterica of 30 different serovars in Germany 2005-11. J Antimicrob Chemother 2013, 68, (9), 1978-81.
5. Park C.H.; Robicsek, A.; Jacoby, G.A.; Daniel Sahm, D.; Hooper, D.C., Prevalence in the United States of aac(6_)-Ib-cr Encoding a Ciprofloxacin-Modifying Enzyme. Antomicrob Agents Chemother, 2006, 50, 3953–3955.
6. Huang, Z.; Mi, Z.; Wang, C., A novel beta-lactamase gene, LAP-2, produced by an Enterobacter cloacae clinical isolate in China. J Hosp Infect 2008, 70, (1), 95-6.

7. Liu, Y. Y.; Wang, Y.; Walsh, T. R.; Yi, L. X.; Zhang, R.; Spencer, J.; Doi, Y.; Tian, G.; Dong, B.; Huang, X.; Yu,L. F.; Gu, D.; Ren, H.; Chen, X.; Lv, L.; He, D.; Zhou, H.; Liang, Z.; Liu, J. H.; Shen, J., Emergence of plasmid mediated colistin resistance mechanism MCR-1 in animals and human beings in China: a microbiological and molecular biological study. Lancet Infect Dis 2016, 16, (2), 161-8.
8. Xavier, B. B.; Lammens, C.; Ruhal, R.; Kumar-Singh, S.; Butaye, P.; Goossens, H.; Malhotra-Kumar, S., Identification of a novel plasmid-mediated colistin-resistance gene, mcr-2, in Escherichia coli, Belgium, June 2016. Euro Surveill 2016, 21, (27).
9. Yin, W.; Li, H.; Shen, Y.; Liu, Z.; Wang, S.; Shen, Z.; Zhang, R.; Walsh, T. R.; Shen, J.; Wang, Y., Novel Plasmid-Mediated Colistin Resistance Gene mcr-3 in Escherichia coli. MBio 2017, 8, (3).
10. Carattoli, A.; Villa, L.; Feudi, C.; Curcio, L.; Orsini, S.; Luppi, A.; Pezzotti, G.; Magistrali, C. F., Novel plasmid-mediated colistin resistance mcr-4 gene in Salmonella and Escherichia coli, Italy 2013, Spain and Belgium, 2015 to 2016. Euro Surveill 2017, 22, (31).
11. Borowiak, M.; Fischer, J.; Hammerl, J. A.; Hendriksen, R. S.; Szabo, I.; Malorny, B., Identification of a novel transposon-associated phosphoethanolamine transferase gene, mcr-5, conferring colistin resistance in dtartrate fermenting Salmonella enterica subsp. enterica serovar Paratyphi B. J Antimicrob Chemother 2017, 72, (12), 3317-3324.
12. Yang, Y.Q.; Li Y.X.; Lei, C.W.; Zhang, A.Y.; Wang, H.N., Novel plasmid-mediated colistin resistance gene mcr-7.1 in Klebsiella pneumoniae. J Antimicrob Chemother. 2018, 73, (7), 1791-1795.
13. Wang, X.; Wang, Y.; Zhou, Y.; Li, J.; Yin, W.; Wang, S.; Zhang, S.; Shen, J.; Shen, Z.; Wang, Y., Emergence of a novel mobile colistin resistance gene, mcr-8, in NDM-producing Klebsiella pneumoniae. Emerg Microbes Infect. 2018, 7, (1):122. doi: 10.1038/s41426-018-0124-z.

Figure 1. S1-nuclease restriction and pulsed-field gel electrophoresis (PFGE): plasmids of donor E. coli isolates from flies and their transconjgants.

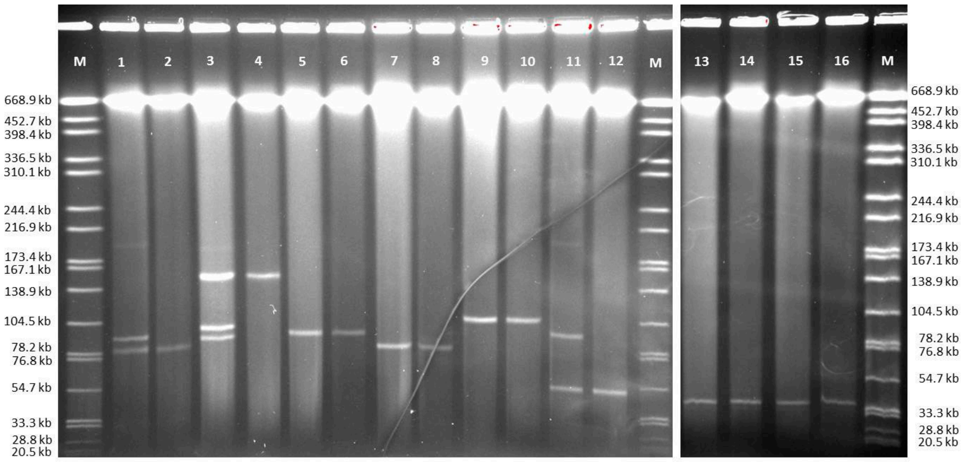

Lane	Sample no. and site	Isolate no.	ESBL	Other β-lactamases	PMQR gene	Phylogenetic group	PFGE-type (clone)	Plasmid sizes
1	20 H	752/17	CTX-M-1	-	-	A	E1	80kb, 90kb
2	-	752/17 K1	CTX-M-1	-	-	A	-	80 kb
3	32 H	754/17	CTX-M-1	TEM	-	A	E3	90kb, 100kb, 160kb
4	-	754/17 K1	CTX-M-1	TEM	-	A	-	160kb
5	61 Z	758/17	CTX-M-1	-	-	A	E5	90kb
6	-	758/17 K1	CTX-M-1	-	-	A	-	90kb
7	62 Z	759/17	CTX-M-3	-	-	D	E6	80kb
8	-	759/17 K1	CTX-M-3	-	-	A	-	80kb
9	79 Z	763/17	CTX-M-1	-	-	D	E9	105kb
10	-	763/17 K1	CTX-M-1	-	-	A	-	105kb
11	157 RB	768/17	CTX-M-1	LAP	*qnrS1*	A	E14	50kb, 90kb
12	-	768/17 K1	CTX-M-1	LAP	*qnrS1*	A	-	50kb
13	31 H	753/17	SHV-12	-	*qnrS1*	A	E2	35kb
14	-	753/17 K1	SHV-12	-	*qnrS1*	A	-	35kb
15	48 H	755/17	SHV-12	-	*qnrS1*	A	E2	35kb
16	-	755/17 K1	SHV-12	-	*qnrS1*	A	-	35kb

Sites: H = hospital, Z = zoo, RA = residential area A, RB = residential area B; PMQR = plasmid mediated quinolone resistance; K1 = transconjugant *E. coli* J53 Azi^{R}; Lane M = *Salmonella* Braenderup H9812 (molecular marker strain, XbaI-restricted).
